# Tunable Electronic Properties of Few-Layer Tellurene under In-Plane and Out-of-Plane Uniaxial Strain

**DOI:** 10.3390/nano12050875

**Published:** 2022-03-06

**Authors:** Genwang Wang, Ye Ding, Yanchao Guan, Yang Wang, Lijun Yang

**Affiliations:** 1 Key Laboratory of Microsystems and Microstructures Manufacturing, Ministry of Education, Harbin Institute of Technology, Harbin 150001, China; nisker@163.com (G.W.); dy1992hit@hit.edu.cn (Y.D.); guanyanchaoo@163.com (Y.G.); 2 School of Mechatronics Engineering, Harbin Institute of Technology, Harbin 150001, China

**Keywords:** tellurene, strain engineering, density functional theory, semiconductor-to-metal transition

## Abstract

Strain engineering is a promising and fascinating approach to tailoring the electrical and optical properties of 2D materials, which is of great importance for fabricating excellent nano-devices. Although previous theoretical works have proved that the monolayer tellurene has desirable mechanical properties with the capability of withstanding large deformation and the tunable band gap and mobility conductance induced by in-plane strain, the effects of in-plane and out-of-plane strains on the properties of few-layer tellurene in different phases should be explored deeply. In this paper, calculations based on first-principles density functional theory were performed to predict the variation in crystal structures and electronic properties of few-layer tellurene, including the α and β phases. The analyses of mechanical properties show that few-layer α-Te can be more easily deformed in the armchair direction than β-Te owing to its lower Young’s modulus and Poisson’s ratio. The α-Te can be converted to β-Te by in-plane compressive strain. The variations in band structures indicate that the uniaxial strain can tune the band structures and even induce the semiconductor-to-metal transition in both few-layer α-Te and β-Te. Moreover, the compressive strain in the zigzag direction is the most feasible scheme due to the lower transition strain. In addition, few-layer β-Te is more easily converted to metal especially for the thicker flakes considering its smaller band gap. Hence, the strain-induced tunable electronic properties and semiconductor-to-metal transition of tellurene provide a theoretical foundation for fabricating metal–semiconductor junctions and corresponding nano-devices.

## 1. Introduction

Since a variety of two-dimensional (2D) materials have been synthesized by mechanical exfoliation, chemical vapor deposition (CVD), physical vapor deposition (PVD), molecular beam epitaxy (MBE), pulsed laser deposition (PLD), and so on [1,2], the mechanical, electrical, optical, and quantum properties make them attractive for applications in transistors, photodetectors, cells, sensors, and memristors [3,4,5,6]. However, despite the promising superiority of 2D materials, their intrinsic properties make them unsuitable for fabricating nano-devices with excellent properties and stability. Hence, methods of tailoring intrinsic properties, such as doping [7,8], electric field tuning [9,10], surface modification [11], and building van der Waals heterostructure [12,13], have become critical ways to improve the performance further. In particular, strain engineering emerges as a perfect candidate for controlling the performance of nano-devices, since the 2D materials are capable of withstanding large in-plane and out-of-plane strains before the rupture [14,15]. Moreover, strain can modulate the mechanical, electrical, and optical properties of 2D materials, like phase stability [16], band structure [17], mobility [18], Fermi level [19], and thermal conductivity [20], so that the strain-induced effects provide a fertile library for controlling the properties of advanced nano-devices. For example, the local strain field, which is generated from engineering the substrate’s surface morphology, leads to a two-orders-of-magnitude increase in the carrier mobility of MoS_2_ transistors, compared to the conventional devices [21]. Tensile strains in monolayer (ML) MoS_2_ dramatically enhanced the sensitivity and response performance of a sensor for gas detection by tuning the Schottky barrier height [22]. In addition, the deformation of 2D materials also introduces new phenomena, like piezotronics and piezophototronics, which have potential applications in energy harvesting, flexible, and self-powered nano-devices [23]. Therefore, the exploration of strain engineering in 2D materials is promising and urgent for future multifunctional nano-devices.

Recently, the existence of 2D tellurium (tellurene) has been predicted by first-principles density functional theory (DFT) calculations [24,25,26]. These results show that there are more than five stable or meta-stable phases for tellurene, which exhibit semiconductor and metallic properties. Of these, at least two phases, α-Te and β-Te (since the names of different phases in different papers are still not unified, the definition of tellurene phases in this paper follows Refs. [25,26]) were synthesized by solution-grown methods [27], liquid-phase exfoliation [28], MBE [29], PLD [30], and PVD [31]. The tellurene has demonstrated high carrier mobility, high on/off ratio, considerable responsivity, and excellent air stability for applications in FET, photodetectors, and sensors [27,32,33]. Tellurene is also a promising candidate for strain engineering. For instance, according to DFT calculations, ML β-Te has a low Young’s modulus (~27 GPa) and large tensile strain limit (>30%), suggesting that it is a kind of ductile material with the capability of withstanding large stretch deformation [34,35]. Moreover, the band structure and mobility of ML β-Te can also be tuned by the strain [35,36]. Experiments also confirmed the strain-induced effects on the electrical properties of tellurene and proved the capacity for developing flexible devices [37,38,39]. Recent calculation works mainly focused on the in-plane deformation of ML β-Te. However, the ML tellurene is still hardly acquired by mechanical exfoliation and solution-grown method for the purposes of experiments, compared to graphene, phosphorene, and hexagonal boron nitride. Therefore, the effects of strain on few-layer and multi-layer tellurene, including the crystal structures and mechanical and electrical properties, should be investigated for the design of nano-devices. Furthermore, strain-effects on different phases also offer diverse ways to select suitable materials and methods of controlling properties. Besides the in-plane strain, the out-of-plane strain induced by normal strain and bending deformation also provides another unique means to design and control the electron transport and electron–phonon coupling process [40,41]. Thus, a deeper insight into the profound impact of in-plane and out-of-plane strain on few-layer Te is indispensable and promising for next-generation nano-devices.

In this work, the strain engineering in few-layer α-Te and β-Te was investigated theoretically. First of all, the crystal structures of few-layer α-Te and β-Te were acquired from the bulk tellurium. Calculation results show the main difference between the two phases is the location of the mid-Te atoms. Then, the mechanical properties of unstrained tellurene were calculated, including Young’s modulus (YM) and Poisson’s ratio (PR). The elasticities of the few-layer α-Te and β-Te were discussed. In addition, the phase transition between the few-layer α-Te and β-Te under the uniaxial strain was investigated by calculating the location of mid-Te atoms. The intrinsic band structures of the few-layer α-Te were also calculated to show the variations as the thickness increases. Sequentially, the strain engineering of the electronic properties of tellurene was performed by gradually applying uniaxial strain in the armchair (AC), zigzag (ZZ), and normal (NM) directions. The semiconductor-to-metal transition (SMT) in tellurene was shown during some deformation processes. Therefore, the variation in band structures and density of states under the strain were calculated to reveal the mechanism of the process of SMT. The calculation results in this paper provide a foundation to design promising nano-devices based on tellurene.

## 2. Methods

All calculations were performed by QUANTUM ESPRESSO based on first-principles density functional theory (DFT) [42,43]. The generalized gradient approximation of the Perdew–Burke–Ernzerhof (PBE) exchange correlation functional and projector augmented-wave (PAW) pseudopotential were adopted [44]. The cut-off energy was set to 500 eV after testing different values. In order to avoid the interaction between flakes and its periodic images, the vacuum space of 20 Å was introduced along the out-of-plane direction. All structures were relaxed until the energy in electronic self-consistent field (SCF) iterations and the ionic Hellmann–Feynman forces were lower than 1 × 10^−6^ eV/atom and 0.01 eV/Å, respectively. In addition, the van der Waals correction proposed by Grimme [45] was considered. During all the calculation processes, a dense k-mesh of 11 × 15 × 1 sampling was used. For the calculations of electronic properties, the spin–orbit coupling (SOC) effect was also included to obtain accurate band structures. To simulate the uniaxial in-plane and out-of-plane deformations of few-layer Te, the tensile and compressive strain were gradually exerted with a step of 1% in the AC, ZZ, and NM directions. For in-plane strain, the lattice constants were fixed in the out-of-plane and strained directions and atoms were fully relaxed in all directions. During the deformation in the NM direction, the movements of the top and bottom atoms were fixed in the NM direction and relaxed in other directions, while other atoms were relaxed in all directions. Meanwhile, the lattice constant in the NM direction was fixed and the lattice constants along in-plane directions were relaxed.

In order to reveal the structural stability of few-layer tellurene with different phases, the formation energy Δ*E* was calculated. The equation was defined as:

(1) $$\Delta E=E_{\mathrm{tellurene}-\mathrm{energy}/\mathrm{atom}}-E_{\mathrm{bulk}-\mathrm{energy}/\mathrm{atom}}$$

where *E*_tellurene-energy/atom_ and *E*_bulk-energy/atom_ are the total energy per atom of tellurene and bulk tellurium, respectively.

To investigate the mechanical properties, the orientation-dependent YM *E* (*θ*) and PR *v* (*θ*) were calculated as [46]

(2) $$\left\{ \begin{array}{l} E(\theta )=\frac{Y_{ZZ}}{\cos^{4}(\theta )+d_{2}\cos^{2}(\theta )\sin^{2}(\theta )+d_{3}\sin^{4}(\theta )}\\ v(\theta )=\frac{v_{ZZ}\cos^{4}(\theta )-d_{1}\cos^{2}(\theta )\sin^{2}(\theta )+v_{ZZ}\sin^{4}(\theta )}{\cos^{4}(\theta )+d_{2}\cos^{2}(\theta )\sin^{2}(\theta )+d_{3}\sin^{4}(\theta )} \end{array}\right.$$

where *d*_1_, *d*_2_, *d*_3_, *Y*_ZZ_, and *v*_ZZ_ are elastic constants, for 2D materials, which are defined as

(3) $$\left\{ \begin{array}{l} v_{ZZ}=\frac{C_{12}}{C_{22}},\;\;\;\;\;Y_{ZZ}=\frac{C_{11}C_{22}-C_{12}^{2}}{C_{22}}\;\;\;\\ d_{1}=\frac{C_{11}}{C_{22}}+1-\frac{C_{11}C_{22}-C_{12}^{2}}{C_{22}C_{66}},\;\;\;\;\;\;d_{2}=-\left(2\frac{C_{12}}{C_{22}}-\frac{C_{11}C_{22}-C_{12}^{2}}{C_{22}C_{66}}\right),\;\;\;\;\;d_{3}=\frac{C_{11}}{C_{22}} \end{array}\right.$$

where *C*_11_, *C*_12_, *C*_22_, and *C*_66_ are elastic stiffness constants, which can be obtained based on the strain energy and Hooke’s law [47], shown as:

(4) $$\left[\begin{array}{l} \sigma_{11}\\ \sigma_{22}\\ \sigma_{12} \end{array}\right]=\left[\begin{array}{l} C_{11}\;\;\;\;\;\;\;\;\;\;\;\;C_{12}\;\;\;\;\;\;\;\;\;\;\;\;0\\ C_{12}\;\;\;\;\;\;\;\;\;\;\;\;C_{22}\;\;\;\;\;\;\;\;\;\;\;\;0\\ \;\;0\;\;\;\;\;\;\;\;\;\;\;\;\;\;\;\;0\;\;\;\;\;\;\;\;\;\;\;\;\;C_{66} \end{array}\right]\left[\begin{array}{l} \varepsilon_{11}\\ \varepsilon_{22}\\ 2\varepsilon_{12} \end{array}\right]$$

(5) $$C_{ij}=\frac{1}{A\left(\varepsilon_{ij}\right)}\left(\frac{\partial E^{2}}{\partial \varepsilon_{i}\partial \varepsilon_{j}}\right)$$

where *E* is the strain energy and *A* (*ε*_ij_) is the area under the strain. *ε* is the applied strain, which is defined as *ε* = (*a* − *a*_0_)/*a*_0_, where *a* and *a*_0_ are the lattice constants of strained and un-strained structures, respectively. The range of applied strain for calculating elastic stiffness constants is from −2% to 2% with a step of 0.5%.

## 3. Results and Discussion

### 3.1. Crystal Structures

**Figure 1:**
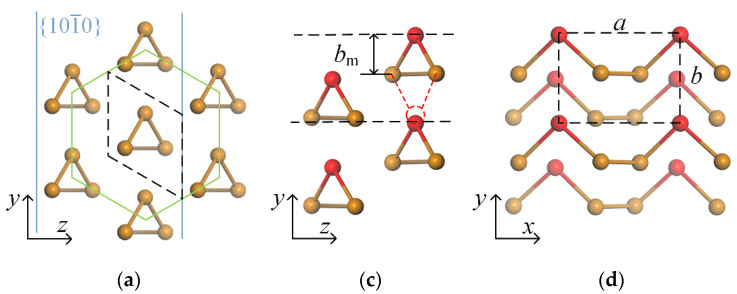

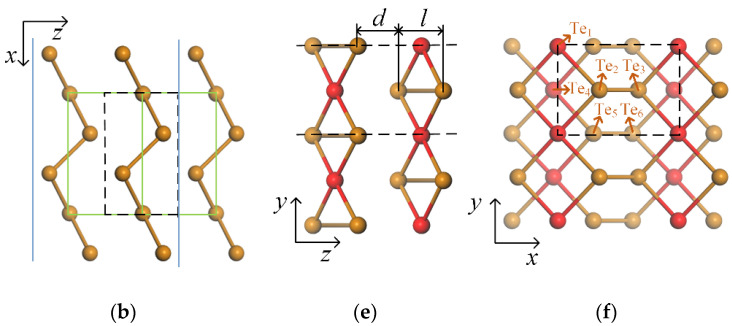
Atomic configurations of the bulk Te, BL α-Te, and BL β-Te. Top (**a**) and side (**b**) views of bulk Te. The green and blue solid lines in (**a**,**b**) show the hexagonal element cell and {10$\overset{-}{1}$0} planes of bulk Te, respectively. Side (**c**) and top (**d**) views of BL α -Te, side (**e**) and top (**f**) views of BL β-Te. *a* and *b* are the lattice constants along the *x*- and *y*-directions. *l* and *d* represent the average of layer thickness and interlayer distance, respectively. The red dotted lines and circles show the symmetric position of the red Te atom and bonds in the β phase. The black dotted lines in (**a**–**f**) show the unit cell in the DFT calculations.

The bulk tellurium, which belongs to the space group P3_1_21, has helical chains of Te atoms that are stacked together by non-covalent bonding at the center and corners of the hexagonal cell. Figure 1a,b present the top and side views of the atomic configurations. The optimized lattice constants of the bulk Te are *a* = *b* = 4.34 Å, *c* = 6.03 Å, which are consistent with previous works [24]. Then, for few-layer α-Te, for instance, the bilayer (BL) shown in Figure 1c,d, can be obtained by cutting the bulk Te along the parallel {10$\overset{-}{1}$0} planes (the blue solid lines in Figure 1a,b). In the α-Te, the Te atoms still maintain the chain structures along the *x*-direction. However, compared to α-Te, the β phase can be regarded as a kind of symmetric structure by moving the mid-Te atoms (the red atoms Te_1_ and Te_4_) to the symmetric position (red dotted circles), presented in Figure 1e,f. The mid-atoms are not only bonded to the atoms in same chains but also the adjacent chains. Therefore, the main difference between the α-Te and β-Te is the locations of the mid-Te atoms. Hence, in this paper, the fractional distance, which was defined as *r* = *b*_m_/*b*, was used to indicate the α and β phases. *b*_m_ is the average distance along the *y* axis between the mid-Te atom and other two atoms in the same Te chain, as shown in Figure 1c. For the β-Te, the mid-Te atoms are located at the symmetric position, so that the fractional distance *r* equals 0.5. However, the α-Te has an *r* value less than 0.5 since the mid-Te atoms are far from the adjacent chains. The lattice parameters and formation energy Δ*E* of α-Te and β-Te with BL, three-layer (TL), and four-layer (FL) are presented in Table 1. According to the crystal structures of ML Te, the α phase is not stable and transforms into β. This phenomenon has been concluded by other theoretical works [25,26]. For the few-layer tellurene, crystal structures of both α and β phase were acquired. Despite the positive formation energy, which means the bulk tellurium is more stable than layered, the relative lower values (for instance, the formation energy of MoS_2_, silicene, and germanene are 0.28 eV/atom, 0.76 eV/atom, and 0.99 eV/atom, respectively [48,49]) also give support to the existence of few-layer α-Te and β-Te, especially for the thicker flakes. The previous experiments and phonon spectra in calculations have also proved the existence of both phases [25,26,27,28,29,30,31]. In addition, the small differences in formation energy between the α and β phases mean the possibility of transition between them. This phase transition has also been predicted by calculation works [50,51]. Hence, due to the differences in the crystal structures, the mechanical and electronic properties of few-layer α-Te and β-Te and the strain engineering in them should be investigated separately.

### 3.2. Mechanical and Electronic Properties

**Figure 2:**
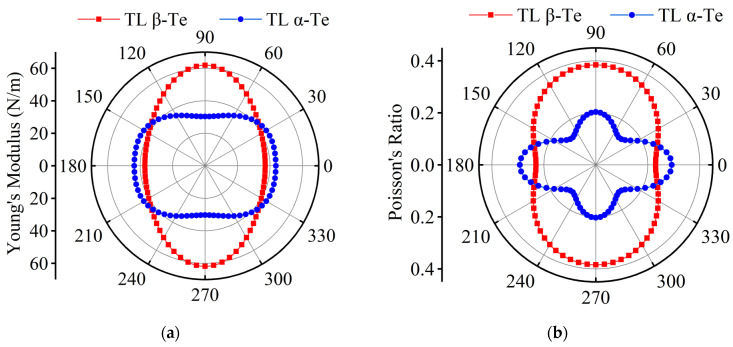
Mechanical properties of TL α-Te and β-Te. (**a**) Orientation-dependent YM, (**b**) orientation-dependent PR.

Before exploring the strain effect on the properties of few-layer α-Te and β-Te, the mechanical properties were firstly glimpsed, including elastic stiffness constants, YM, and PR. Table S1 shows the calculated results. For ML and few-layer Te, the elastic stiffness constants satisfy the equilibrium conditions (*C*_11_*C*_22_-*C*_12_^2^ > 0, and *C*_11_, *C*_66_ > 0), which proves the mechanical stability of all crystal structures. Figure S1 presents the orientation-dependent YM and PR of ML β-Te. The maximal YM is 24.8 N/m along the ZZ direction and the minimal is 11.1 N/mm for the AC direction. The corresponding PR values are 0.59 and 0.26 along the ZZ and AC directions, respectively. These results, which are similar to the previous works [34,35], indicate that the ML β-Te has a stronger anisotropy of mechanical properties and is more easily deformed in the AC direction than in the ZZ direction. However, for few-layer tellurene, α and β phases exhibit significant differences in YM and PR. Figure 2 shows the orientation-dependent YM and PR of TL tellurene (the BL and FL tellurene are shown in Figure S2). In order to investigate the effect of uniaxial strain on electronic properties, YM and PR in the direction of AC (*θ* = 0°) and ZZ (*θ* = 90°) are mainly focused upon. Except for the BL flakes, α-Te has smaller YM and PR along the ZZ direction than AC, which can be attributed to the bonding atom chains in the AC direction but weak van-der-Waals-type bonds between chains (ZZ direction), as shown in Figure 1c,d. The performance of few-layer β-Te is analogous to ML β-Te. Compared to α-Te, the values of YM and PR are slightly decreased along the AC direction. However, few-layer β-Te has larger YM and PR along the ZZ direction. Referring to the crystal structures shown in Figure 1e,f, mid-Te atoms are not only bonded to other atoms in same chains but also the atoms in adjacent chains in the β phase, so that deformation along the ZZ direction requires a larger force to overcome the bond energy. Conclusively, few-layer α-Te and β-Te have inverse anisotropic elastic properties, which are dominated by the location of mid-Te atoms. However, α-Te is more sensitive to the strain than β-Te in the ZZ direction, which is beneficial for tuning electrical properties by strain.

**Figure 3:**
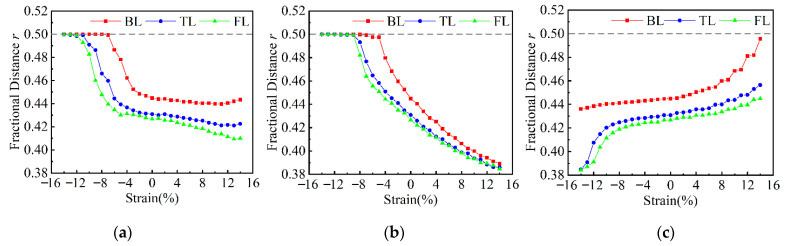
Fractional distances of few-layer α-Te versus uniaxial strain in different directions. (**a**) AC direction; (**b**) ZZ direction; (**c**) NM direction.

As mentioned in other calculation works, the transition between the α and β phases is feasible [26,50]. To give an insight into the phase transition, the variations in atom structures under uniaxial strain in the range −14% ≤ *ε* ≤ 14% were calculated. Despite compressive and tensile strains applied on β-Te in AC, ZZ, and NM directions, the fractional distance *r* is still maintained at 0.5, which means no phase transition from β-Te to α-Te occurs during uniaxial deformation. However, as Figure 3 shows, few-layer α-Te can be transformed to the β phase under compressive strain in the AC or ZZ directions and will maintain the β phase even if the compressive strain increases. The critical strains of phase transition for BL, TL, and FL α-Te are about −7%, −11%, and −13%, respectively, in the AC direction, and −5%, −9%, and −9% in the ZZ direction. The lower critical strain in the ZZ direction is consistent with the lower YM in the ZZ direction and the higher PR in the AC direction, which confirms the capacity of uniaxial strain to change atom structures. In addition, thicker flakes require higher strain for the phase transition due to higher deformation resistibility. When the tensile strain applies along the AC and ZZ directions, the decreased fractional distances indicate the persistence of the α phase, which means no phase transition occurs. For the normal strain shown in Figure 3c, despite BL α-Te approximately transforming to β-Te when the tensile strain attains 14%, fractional distances of TL and FL only increase slightly under tensile strain and no phase transition is shown at least in the range 0 ≤ ε ≤ 14%. Furthermore, the impact of tensile strain on fractional distances becomes smaller as the thickness increases. Hence, according to the results, the compressive strain in the ZZ direction is more suitable to induce the phase transition from α to β due to lower required strain (−5–−9%). Previous calculations have also shown that the strain limits of ML tellurene attain −24% and −22% in the AC and ZZ directions [35]. Although no calculations and experiments have proved the strain limits of few-layer tellurene so far, the compressive strains in the ZZ direction still have the potential to induce phase transition before the fracture of materials. The strain-induced phase transition and the corresponding application should be further confirmed in experiments. In addition, attention should be paid to the phase transition during the tuning of electronic properties by the strain. 

**Figure 4:**
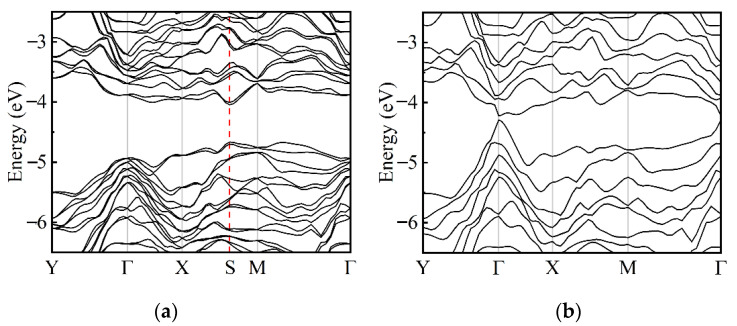
Band structures of TL tellurene. (**a**) α-Te; (**b**) β-Te.

Since the α-Te and β-Te present the different performances of mechanical properties, the electronic properties of these two phases also need to be compared. The Brillouin zone used in the calculations and the results of these are shown in Figure S3; the ML β-Te is a semiconductor 2D material with direct bandgaps of 1.04 eV and 1.46 eV calculated by PBE + SOC and HSE (hybrid functional) +SOC schemes, respectively, which are consistent with previous works [24,35]. The valence band maximum (VBM) and conduct band minimum (CBM) of ML β-Te are both located at the Γ point and determined by the p_y_ and p_x_ orbitals of Te, respectively, and the total density of states (TDOS) and projected density of states (PDOS) are shown in Figure S3d. These suitable direct band gaps reveal that the ML β-Te is a promising 2D material for application in electrical and optical nano-devices. The band structures of few-layer tellurene are shown in Figure 4 and Figure S4. The band gaps of both α and β phases decay as the thickness increases; these are 0.82 eV (BL), 0.62 eV (TL), and 0.46 eV (FL) for α-Te and 0.33 eV (BL), 0.07 eV (TL), and 0.03 eV (FL) for β-Te. Apparently, the β phase has a smaller band gap than α for the same thickness. Moreover, the α-Te with different layers exhibits variation in VBM and CBM. While ML Te has a direct band gap at the point of Γ, BL α-Te shows an indirect band gap as the CBM moves to the point between Γ and X, as shown in Figure S4a. However, the VBM and CBM shift to around the point of S (0.50, 0.32, 0) and the TL and FL α-Te turn into direct semiconductors again. The few-layer β-Te still has a direct band gap at the point of Γ regardless of the thickness. Hence, comparing to the α-Te, the few-layer β-Te is more easily converted to metal due to the small band gap. 

### 3.3. Effects of Uniaxial Strain on Electronic Properties

**Figure 5:**
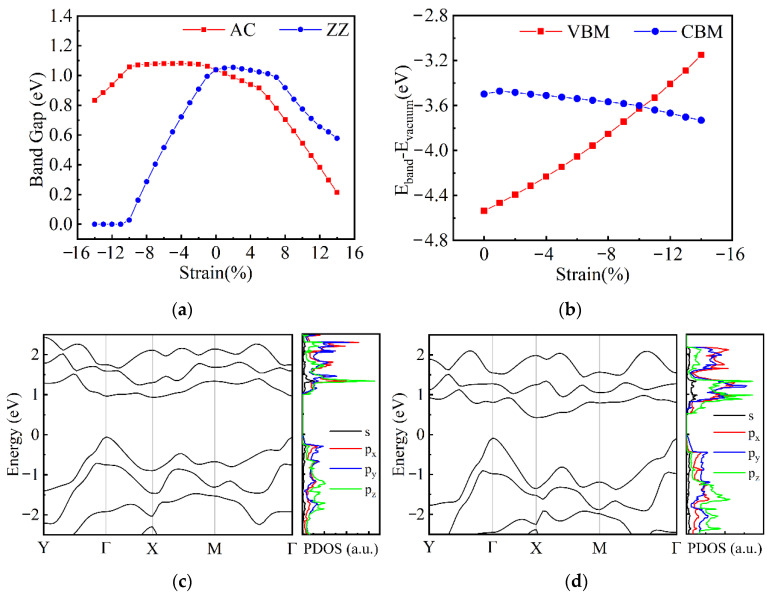
Electronic properties of ML β-Te under the in-plane uniaxial strain. (**a**) The variation in band gap; (**b**) the changes in the energy of VBM and CBM under the compressive strain along the ZZ direction; band structures (left) and PDOSs (right) under the strains of −1% (**c**) and −6% (**d**) along the ZZ direction.

As the α-Te and β-Te show distinct mechanical and electronic properties, performances of these two phases under uniaxial strain should be discussed to explore the control of electronic properties. The strain effects on ML β-Te were firstly investigated. Figure 5a shows the variation in band gap when compressive and tensile strains are exerted on the ML β-Te along the AC and ZZ directions. Under the compressive strains along the AC direction and the tensile strain along the ZZ direction, the band gap of ML β-Te slightly varies at first and then decays quickly after a critical strain (−11% and 7%, respectively). However, the decrease in band gap is still small as the strains increase to −14% and 14%, which means limited effects of these kinds of strain on the band structure. However, when the compressive strain along the ZZ direction applies, the band gap decreases more quickly than strains in the other direction and induces the SMT with a critical strain of −11%. In order to investigate the mechanism of the SMT induced by uniaxial strain, the band structures and PDOS were calculated. Figure 5b presents the variation in the energy of the VBM and CBM (with respect to vacuum level) during the compression process. The compressive strain not only forces the CBM to drop down to a lower energy, but also pushes the VBM up to a higher energy, which results in the SMT. Moreover, the strain has a greater impact on the energy of VBM rather than CBM. Figure 5c,d show the band structures and the PDOSs of ML β-Te when the strain is −1% and −6%. Compared to the intrinsic band structure shown in Figure S3b, the CBM shifts from Γ to X and the direct band gap suddenly becomes indirect even when small compressive strain (−1%) is applied. As the compressive strain increases, the CBM and VBM maintain the location but the gap between them becomes smaller until the transition to metal (see Figure S5). To have a deep insight into the shift of CBM, the PDOSs of ML β-Te under the compressive strain are illustrated in the right diagrams of Figure 5c,d and Figure S5. Compared to the unstrained ML β-Te, the VBM is still determined by the p_y_ orbitals of Te. However, the CBM is mainly contributed by p_z_ instead of p_x_ orbitals. Figure S6 gives the PDOSs of every Te atom in the ML β-Te under compressive strain of −6%. It is apparent that the p_z_ orbitals are contributed from the two middle Te atoms in the unit cell (Te_2_ and Te_2_ in Figure 1f and Figure S3a). Therefore, the compressive strain along the ZZ direction drives the two atoms to move closer to each other, resulting in the overlap of p_z_ orbitals and the shift in CBM. Hence, the compressive strain along the ZZ direction tunes the energy and location of VBM and CBM and enables the semiconducting ML β-Te to convert to metal. Besides the compressive strain along the ZZ direction, the tensile strain along the AC direction also results in a decrease in the band gap. However, no SMT in ML β-Te is observed until the strain increases to 14%. Figure S7 shows the details of band structures in this case. The ML β-Te changes to a semiconductor with an indirect bad gap (the CBM shifts to the point between X and M) when the tensile strain attains 6% and become a metal when the strain increases to 17%. Although the previous calculation works showed that the tensile strain limit of ML β-Te in the AC direction is larger than 36% [34,35], such high strain may cause the plastic deformation of materials and is hardly performed in experiments. Hence, comparing the tensile strain in the AC direction, the compressive strain along the ZZ direction is more suitable for realizing the SMT. However, it should be noted that the compressive strain of −11% is still hard to exert in experiments. Further experimental investigations are indispensable for the application of strain-induced SMT.

**Figure 6:**
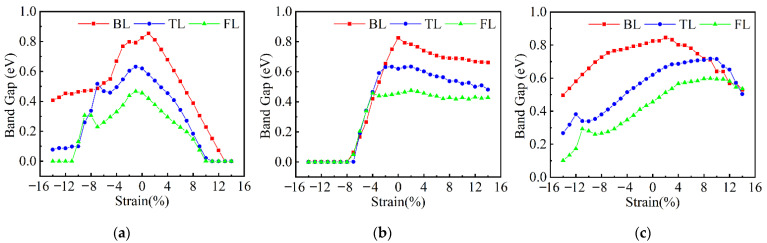
The variation in band gap of few-layer α-Te under uniaxial strain in different directions. (**a**) AC direction; (**b**) ZZ direction; (**c**) NM direction.

The band gaps of few-layer α-Te under uniaxial strain along the AC, ZZ, and NM directions are shown in Figure 6. Similar to the ML β-Te, as the tensile strain applies along the AC direction, the band gap gradually decreases and finally reaches zero. However, the critical strains for the SMT (13%, 11%, and 10% for BL, TL, and FL, respectively) are smaller than MLβ-Te and tend to be lower as the thickness increases. Although large compressive strain along the AC direction also leads to a narrow band gap, only FL α-Te shows SMT in the range −14% ≤ *ε* ≤ 0. Since the thicker α-Te has a lower band gap, it can be concluded that the SMT will also occur when the layer increases. During the deformation along the ZZ direction, the change in the band gap is minor when applying the tensile strain or small compressive strain, especially for TL and FL α-Te. As the compressive strain reaches a certain value, the band gap suddenly decays and disappears quickly. The critical strains for SMT are ~−8% for BL, TL, and FL α-Te, which are lower than the situation of tensile strain along the AC direction. Hence, the compressive strain along the ZZ direction is more suitable for the realization of SMT. Moreover, the required strain for the SMT in few-layer α-Te are smaller than ML tellurene. However, the strain along the NM direction cannot result in SMT at least in the range −14% ≤ *ε* ≤ 14% (the transition strain is −21% for FL α-Te, as shown in Figure S8), despite the decrease in band gap under compressive strain. In addition, small tensile strain along NM can increase the band gap, which provides a way to tune the electrical and optical properties for the application of nano-devices.

**Figure 7:**
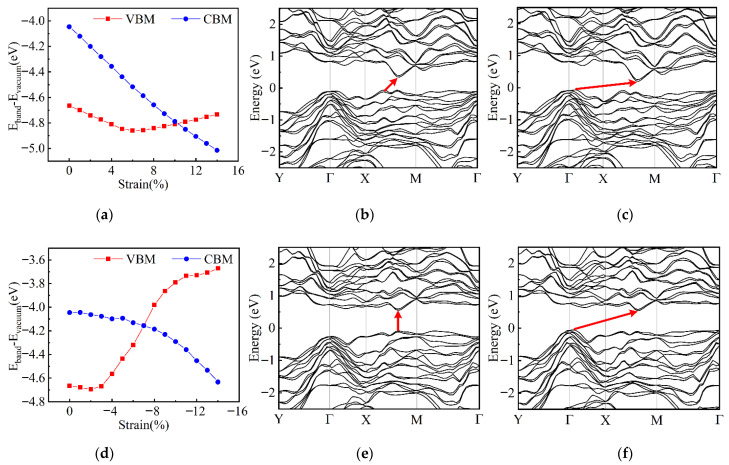
The variation in band structures of TL α-Te under uniaxial strain. The changes in the energy of VBM and CBM with tensile strains along the AC direction (**a**) and compressive strains along the ZZ direction (**d**); band structures of TL α-Te under tensile strains of 5% (**b**) and 7% (**c**) along the AC direction and compressive strains of −1% (**e**) and −3% (**f**) along the ZZ direction.

The effects of tensile strain along the AC direction and compressive strain along the ZZ direction on the band structures of few-layer α-Te are discussed to gain a deeper insight into the mechanism of strain-induced SMT. During the stretching process in the AC direction, the energy of VBM is sensitive to the deformation and drops down linearly, as shown in Figure 7a. The CBM drops down before the strain increases to around 6% and then rises up after that. Therefore, this “drop-rise” process of VBM induces a slow followed by a fast decrease in band gap (see Figure 6a). However, for the compression in the ZZ direction, the changes in the energy of CBM and VBM, shown in Figure 7d, are diametrically opposed to the stretching process in the AC direction. The compressive strain slightly puts the CBM down to lower energy before the occurrence of SMT. The VBM goes up quickly after a minor decline. This variation in VBM results in a lower necessary strain for the realization of SMT by contrasting with the stretching scheme. Figure 7 also shows the band structures of TL α-Te under some critical strain in these two deformation schemes. The unstrained TL α-Te has a direct band gap with CBM and VBM locating at the point of S (0.50, 0.32, 0), as shown in Figure 4b. As the tensile strain applies along the AC direction, the band structure still maintains a direct type at first but the energy of the S point gradually decays relatively. When the tensile strain increases to 5%, the VBM shifts to the point of (0.50, 0.18, 0) and induces an indirect gap, as shown in Figure 7b. Then, the VBM is moved to near the Γ point by further stretching and the energy of it starts to rise up until the occurrence of SMT (see Figure S9a). Therefore, there is a long shift process of VBM with the decrease in energy. In addition, during the whole stretching process, although the strain leads to a large decline in CBM energy, the influence on the location of CBM is small. Figure 7e,f demonstrate the variation in band structure during the compressive strain application. Similar to the tensile strain along the AC direction, the band structures of α-Te under the low compressive strain along the ZZ direction are also the direct type. However, the VBM quickly shifts to around the Γ point when the strain increases to −3% without any transitional location during the process. Before the SMT (see Figure S9b), the locations of VBM and CBM no longer change. However, the energy of VBM dramatically rises, which corresponds to the changes shown in Figure 7d. In addition, unlike the ML β-Te, during the transition from semiconductor to metal, neither scheme can change the dominant orbitals of Te atoms in the CBM and VBM. The VBM has been determined by the p_y_ and the CBM depends on p_y_ and p_z_ orbitals (see Figure S10). Hence, according to the variation in band structure, although the tensile strain along the AC direction and compressive strain along the ZZ direction both induce the SMT, the intrinsic mechanism is different. The compressive strain along the ZZ direction is a preferred candidate for making the band gap narrower or resulting in SMT.

**Figure 8:**
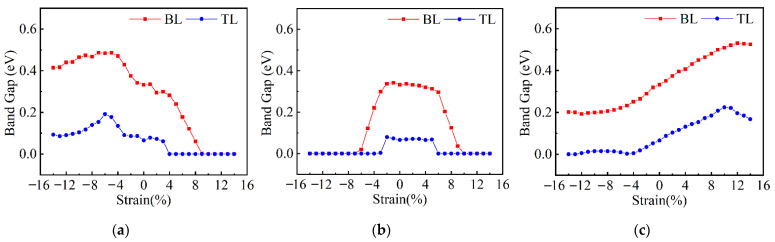
The variation in band gap of few-layer β-Te under uniaxial strain in different directions. (**a**) AC direction; (**b**) ZZ direction; (**c**) NM direction.

**Figure 9:**
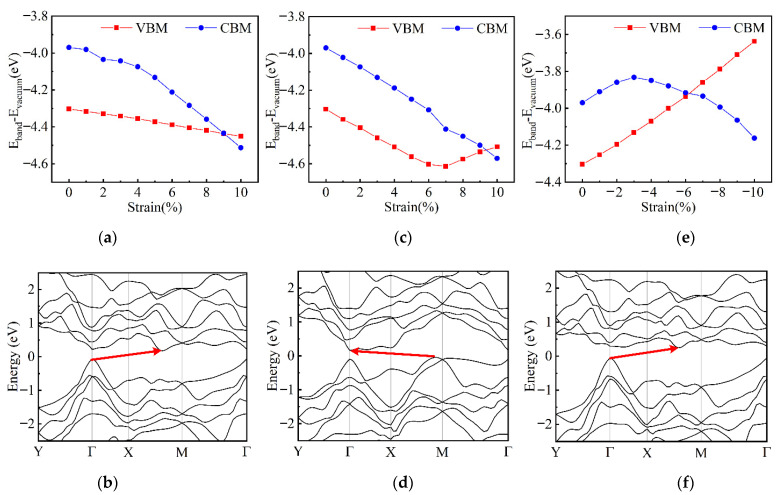
The variation in band structures of BL β-Te under uniaxial strain. The changes in the energy of VBM and CBM with tensile strains along the AC direction (**a**), and tensile (**c**) and compressive (**e**) strains along the ZZ direction. Band structures of BL β-Te under tensile strain of 5% (**b**) along the AC direction, and tensile strains of 7% (**d**) and compressive strain of −3% (**f**) along the ZZ direction.

With a very small band gap, the FL β-Te can easily become metal by applying uniaxial strain, referring to Figure S11. To show the effect of strain on few-layer β-Te, the variations in band gap in BL and TL β-Te under uniaxial strain were plotted in Figure 8. For the in-plane deformation, except the slight enlargement of band gap induced by small compressive strain along the AC direction, the tensile strain along the AC direction and the deformation in the ZZ direction will result in the SMT. It is apparent that the required strain for SMT is lower for thicker tellurene. To explore the mechanism of SMT in few-layer β-Te, the BL β-Te is chosen as an example. Changes in band structures induced by uniaxial strain are shown in Figure 9. The tensile strain along the AC direction slightly puts the VBM down to lower energy and cannot change the location of it. However, the CBM shifts from the location of Γ to X with a minor reduction in energy, when the tensile strain increases to 5%, as shown in Figure 9b. The VBM energy decays fast with no changes in the location until the occurrence of SMT, as the strain reaches 10% (the band structure is shown in Figure S12a). For the deformation in the ZZ direction, the direct band gap changes to indirect due to little shift of CBM when small tensile and compressive strains apply, as shown in Figure S13. As the tensile strain increases, both the energy of CBM and VBM similarly drop, which indicates that the band gap has a small variation. However, when the strain reaches 7%, as the Figure 9d shows, the VBM moves to the point between *X* and *M*. After that, the energy of VBM starts to increase, which leads to the SMT (the band structure under the critical strain of 10% is shown in Figure S12b). The effect of compressive strain on the band structures is similar to the tensile strain. However, the compressive strain raises the energy of CBM and VBM initially and then puts the CBM down after the location shifts to around the *S* points (Figure 9f). Comparing the other two schemes, the required compressive strain in the ZZ direction for the SMT is the lowest. The band structure of the metric BL β-Te induced by compressive strain of −7% is shown in Figure S12c. In addition, the band structure is extremely similar to the BL α-Te under the strain of −8%. This can be attributed to the phase transition from α to β. Therefore, the phase transition also has effects on the electric properties during the uniaxial deformation. The variations in PDOSs are shown in Figure S15. The CBM and VBM of intrinsic BL β-Te depend on the p_y_ orbitals of Te atoms. In the three deformation methods, only the tensile strain along the ZZ direction has a significant influence on the CBM and makes the primary PDOS change to p_x_, which can be attributed to the strain-induced overlap of p_x_ orbitals of the four Te atoms (Te_1,_ Te_2,_ Te_4,_ and Te_6_ shown in Figure 1f); see Figure S16. Under the strain in the NM direction, the few-layer β-Te exhibits a similar performance to α-Te. The tensile strain is capable of enlarging the band gap and the compressive strain can hardly induce the SMT, despite slightly decreasing the gap. Hence, due to the lower required strain, the compressive strain in the ZZ direction is also the most applicable way to induce the SMT. 

The summary of the effects of in-plane and out-of-plane uniaxial strain on the band gap of tellurene is shown in Table 2. Compared to the ML tellurene, the few-layer tellurene is more easily converted to metal. For both α and β phases, the compressive deformation in ZZ is the most suitable to induce the SMT due to the lower critical strain. Moreover, it should be noted that the required compressive strain for the SMT in β-Te is smaller than α-Te with the same layer, especially for thicker flakes. Hence, although the β-Te is hard to deform in the AC direction due to the higher YM, it becomes a metal more easily than the α-Te. This indicates that a preprocess of converting α to β tellurene by other methods, like charge doping, is also a promising way to prompt the SMT. The strain-induced SMT in few-layer tellurene provides a fertile library for tuning the performance of materials, building metal–semiconductor junctions, and fabricating corresponding transistors. Besides the SMT, the capacity of enlarging the band gap of few-layer tellurene showed by the strain in NM direction is also a promising way to modify the properties of nano-devices. 

## 4. Conclusions

In summary, the mechanical and electronic properties of α and β tellurene under uniaxial strain were investigated by first-principles density functional theory calculations. The calculation results concerning the crystal structures reveal that the main difference between the α-Te and β-Te is the location of the mid-Te atoms. The mid-Te atoms in β-Te locate at the symmetrical position and are bonded to the atoms in adjacent chains. Due to the bonds between the two Te atom chains, the β-Te exhibits dramatic higher YM and PR in the ZZ direction and similar performance in the AC direction, compared to the α-Te. Hence, the α-Te is more easily deformed by uniaxial strain in the ZZ direction. In addition, the variation in the atoms’ configuration under uniaxial strain has showed that the in-plane compressive strain can induce the phase transition from α to β. However, no reverse transition is presented during in-plane and out-of-plane uniaxial deformation processes. For the instinct band structures, the few-layer α-Te and β-Te have narrower band gaps as the layer increases, but the band gap of β-Te decays more dramatically than the α-Te. Sequentially, the effects of uniaxial strain (in the range −14% ≤ *ε* ≤ 14%) on electronic properties were investigated. For the ML tellurene, the in-plane deformation induces the decrease in band gap. The layer compressive strain in the ZZ direction can induce the SMT. The strain-induced SMT was also observed in few-layer α-Te and β-Te. Among different in-plane and out-of-plane deformations, compression in the ZZ direction is the most suitable approach to convert the tellurene to metal. The required transition strain in few-layer tellurene is lower than in ML and becomes lower as the thickness increases, especially for β-Te. The strain-induced SMT in tellurene provides a novel way to build metal–semiconductor junctions or even nano-devices in the same flakes without other metal electrode materials. In addition, although the normal strain is incapable of the SMT, the ability to enlarge the band gap greatly by tensile strain also has significant applications in controlling the performance of nano-devices. Therefore, strain-induced tuning of band structures in few-layer tellurene provides a foundation to design FETs, photodetectors, sensors, and memristors.

## Figures and Tables

**Table 1:** Lattice parameters and formation energy Δ*E* of α-Te and β-Te.

Phase	Layer Number	*a* (Å)	*b* (Å)	*l* (Å)	*d* (Å)	*r*	Δ*E* (eV/atom)
α-Te	ML	5.49	4.17	2.16	/	0.50	0.228
	BL	5.80	4.24	2.10	1.79	0.43	0.134
	TL	5.89	4.28	2.07	1.77	0.42	0.095
	FL	5.92	4.30	2.08	1.75	0.42	0.073
β-Te	ML	5.49	4.17	2.16	/	0.50	0.228
	BL	5.78	4.18	2.1	1.88	0.50	0.137
	TL	5.89	4.18	2.05	1.87	0.50	0.101
	FL	5.94	4.19	2.04	1.83	0.50	0.081

**Table 2:** Effects of in-plane and out-of-plane uniaxial strain on the band gap of tellurene.

Phase	Layer Number	AC Direction		ZZ Direction		NM Direction	
		Tensile	Compressive	Tensile	Compressive	Tensile	Compressive
α-Te	BL	SMT at 13%	decrease	slight	SMT at −8%	decrease	decrease
	TL	SMT at 11%	decrease	slight	SMT at −7%	decrease	increase then decrease
	FL	SMT at 10%	SMT at −11%	slight	SMT at −8%	decrease	increase then decrease
β-Te	ML	decrease	decrease	decrease	SMT at −11%	decrease	/
	BL	SMT at 10%	increase then decrease	SMT at 10%	SMT at −7%	decrease	increase then decrease
	TL	SMT at 4%	increase then decrease	SMT at 6%	SMT at −4%	decrease	increase then decrease

## Data Availability

The data presented in this study are available on request from the corresponding author.

## Supplementary Materials

The following are available online at https://www.mdpi.com/article/10.3390/nano12050875/s1, Table S1: Elastic stiffness constants, minimal and maximal YM and PR of α-Te and β-Te, Figure S1: Mechanical properties of ML β- Te, Figure S2: Mechanical properties of BL and FL tellurene, Figure S3: Band structures of ML β-Te, Figure S4: Band structures of BL and FL tellurene, Figure S5: Band structures (left) and PDOSs (right) under the strains of −11% along the ZZ direction, Figure S6: PDOS of Te atoms in ML β-Te, Figure S7: Band structures of ML β-Te under tensile strain along the AC direction, Figure S8: Band structures of FL β-Te under compressive strain of −21% along the NM direction, Figure S9: Band structures of metallic TL α-Te induced by uniaxial strains, Figure S10: PDOS of TL α-Te under uniaxial strain, Figure S11: Band structures of metallic FL β-Te induced by uniaxial strain, Figure S12: Band structures of metallic BL β-Te induced by uniaxial strain, Figure S13: Band structure of BL β-Te under small uniaxial strain along the ZZ direction, Figure S14: Band structure of BL α-Te under compressive strain of −8% along the ZZ direction, Figure S15: PDOSs of BL β-Te under uniaxial strain, Figure S16: PDOS of Te atoms in BL β-Te under tensile strains of 7% along the ZZ direction.
