# Supplementary material for: Tunable Electronic Properties of Few-Layer Tellurene under In-Plane and Out-of-Plane Uniaxial Strain
Source: Nanomaterials (Basel). 2022 Mar 6;12(5):875. doi: 10.3390/nano12050875 (PMC8912431; doi:10.3390/nano12050875)
Supplement: Supplementary file 1 [file nanomaterials-12-00875-s001.zip › nanomaterials-1618738-supplementary.pdf]

Supplementary Materials

Tunable Electronic Properties of Few-Layer Tellurene Under In-Plane and Out-of-plane Uniaxial Strain

Genwang Wang[1,2], Ye Ding [1,2], Yanchao Guan[1,2], Yang Wang[1,2,] *, and Lijun Yang[1,2,]*

[1] Key Laboratory of Microsystems and Microstructures Manufacturing, Ministry of Education, Harbin Institute of Technology, Harbin 150001, China; nisker@163.com (G.W.); dy1992hit@hit.edu.cn (Y.D.); guanyanchaoo@163.com (Y.G.)

[2] School of Mechatronics Engineering, Harbin Institute of Technology, Harbin 150001, China

* Correspondence: wyyh@hit.edu.cn (Y.W.); yljtj@hit.edu.cn (L.Y.)

Table S1. Elastic stiffness constants, minimal and maximal YM and PR of α-Te and β-Te

Phase	Layer Number	C_{11} (N/mm)	C_{12} (N/mm)	C_{22} (N/mm)	C_{66} (N/mm)	E_{min} (N/mm)	E_{max} (N/mm)	υ_{min}	υ_{max}
α	BL	24.8	26.1	3.7	10.1	23.7	25.6	0.14	0.18
	TL	46.4	32.2	9.4	17.8	30.3	44.0	0.14	0.29
	FL	65.7	41.9	14.8	26.0	38.6	62.9	0.11	0.35
β	ML	13.2	29.4	7.8	6.6	11.1	24.8	0.26	0.59
	BL	17.4	46.9	7.0	9.4	16.4	44.1	0.15	0.40
	TL	40.7	67.9	15.6	16.8	37.1	61.9	0.23	0.38
	FL	65.5	89.5	24.2	21.9	58.2	80.6	0.27	0.40

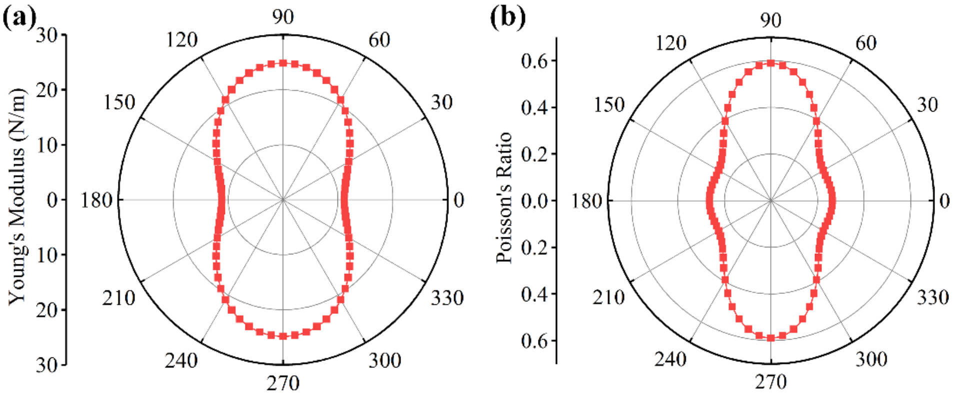

Figure S1 Mechanical properties of ML β- Te. (a) Orientation-dependent Young's modulus (b) Orientation-dependent Poisson's ratio

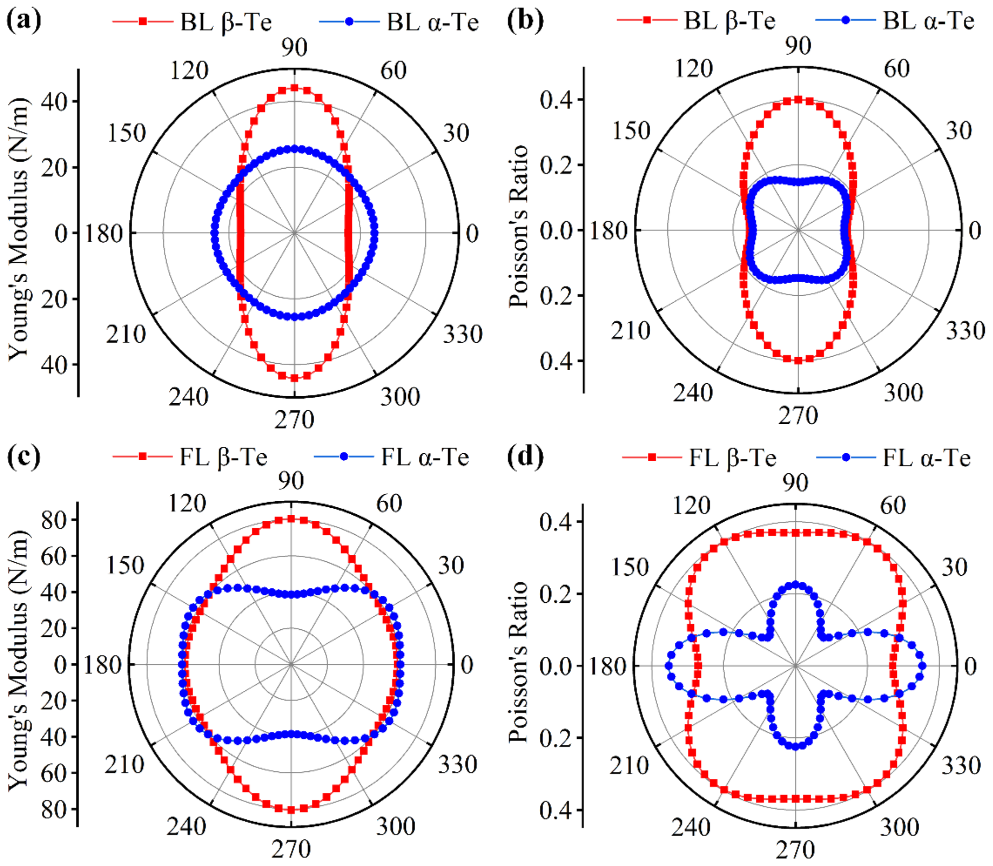

Figure S2. Mechanical properties of BL and FL tellurene. Orientation-dependent Young's modulus of BL (a) and FL (c) Te; Orientation-dependent Poisson's ratio of BL (b) and FL (d) Te.

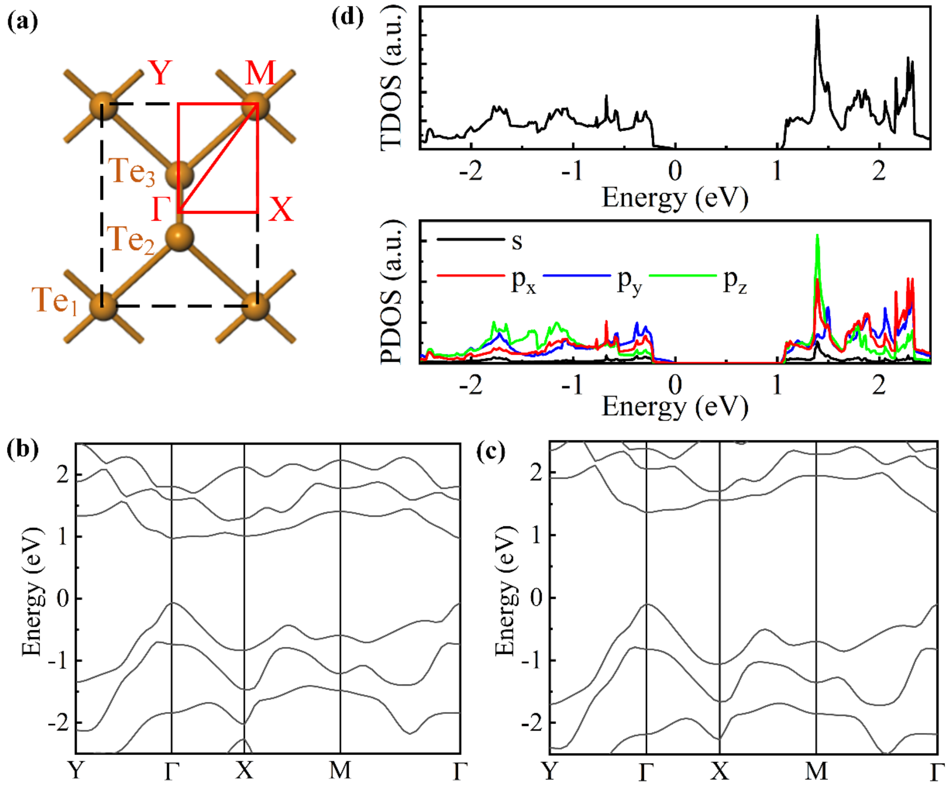

Figure S3. Band structures of ML β-Te. (a) Surface Brillouin zone; Band structures obtained within PBE+ SOC (b) and HSE + SOC schemes (c); (d) TDOS and PDOS;

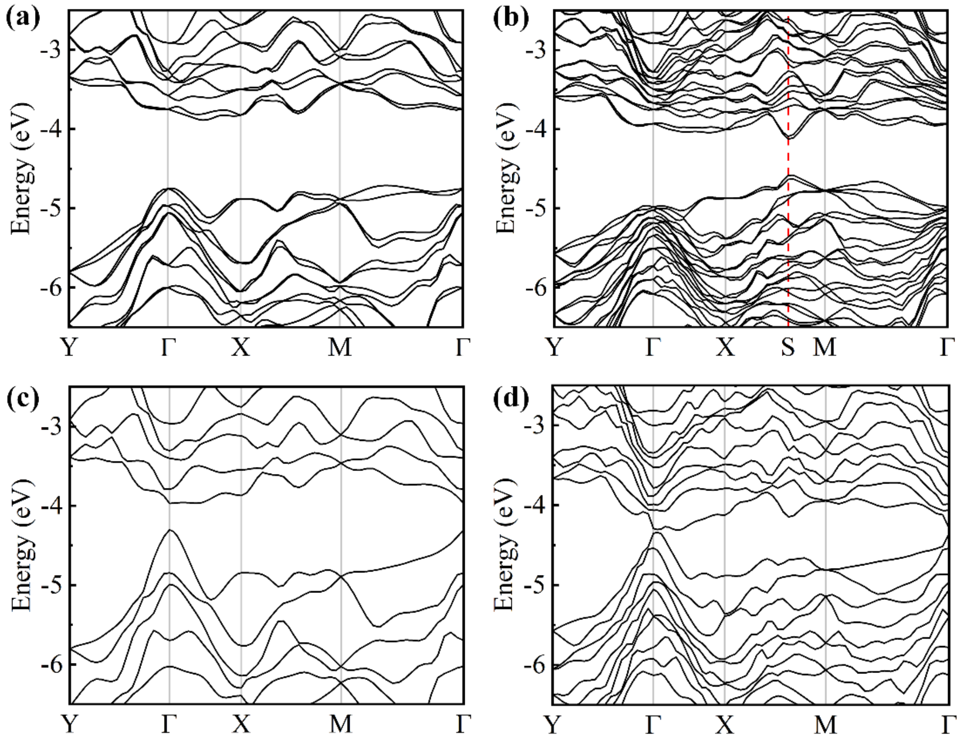

Figure S4. Band structures of BL and FL tellurene. (a) BL α-Te; (b) BL β-Te; (c) FL α-Te; (d) FL β-Te.

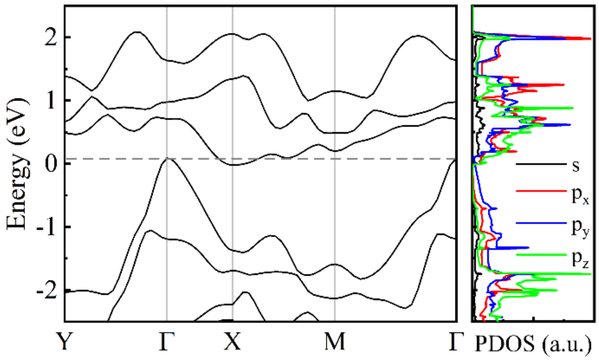

Figure S5. Band structures (left) and PDOSs (right) under the strains of -11% along the ZZ direction;

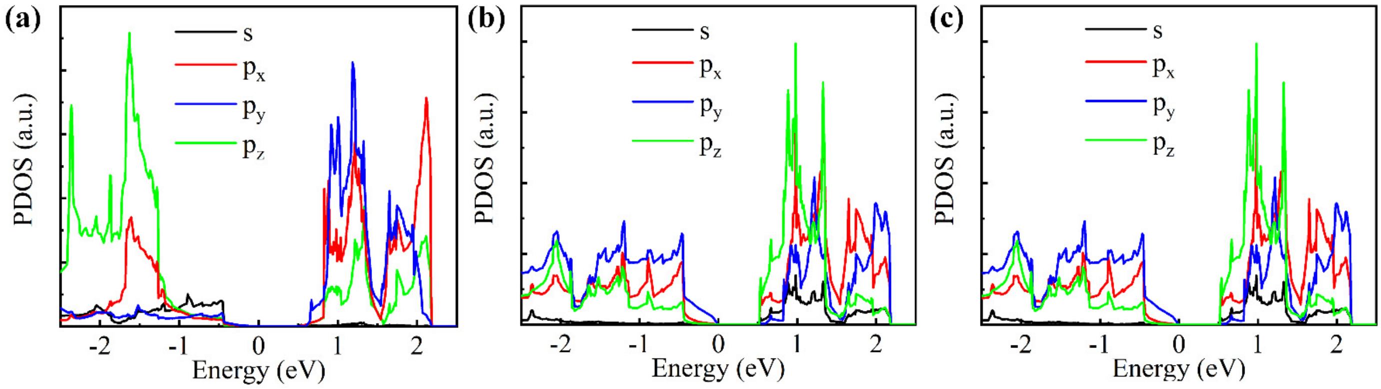

Figure S6. PDOS of Te atoms in ML β-Te. (a) Te_1; (b) Te_2; (c) Te_3.

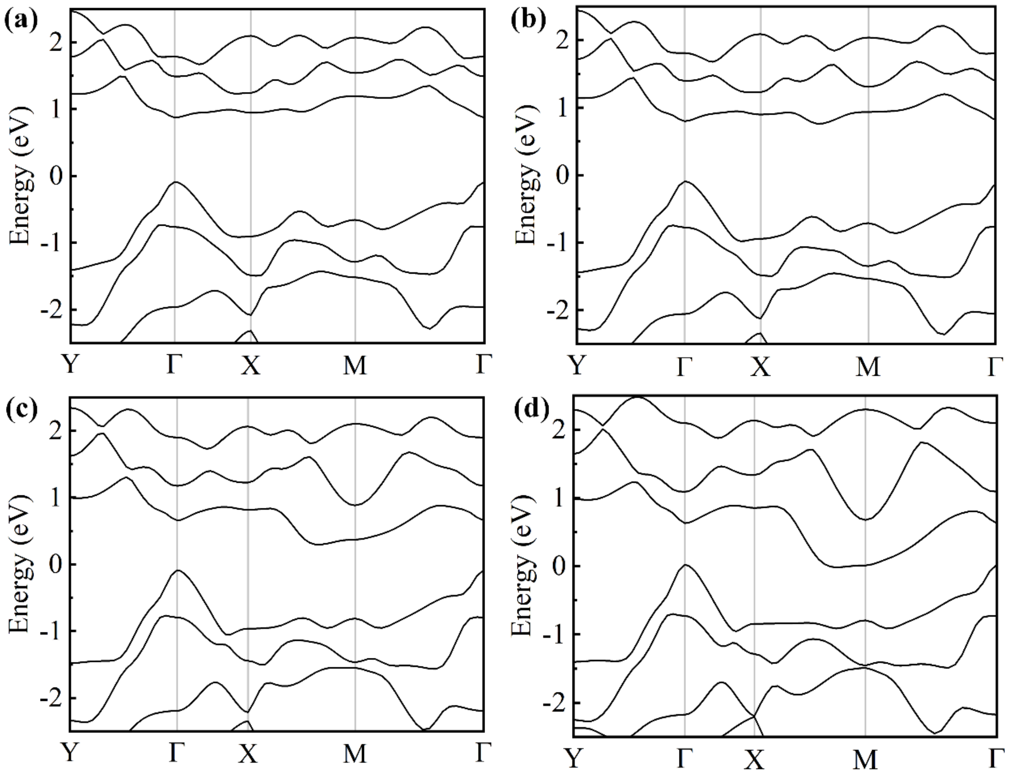

Figure S7. Band structures of ML β-Te under tensile strain along the AC direction. (a) 3%; (b) 6%; (c) 12%; (d) 17%.

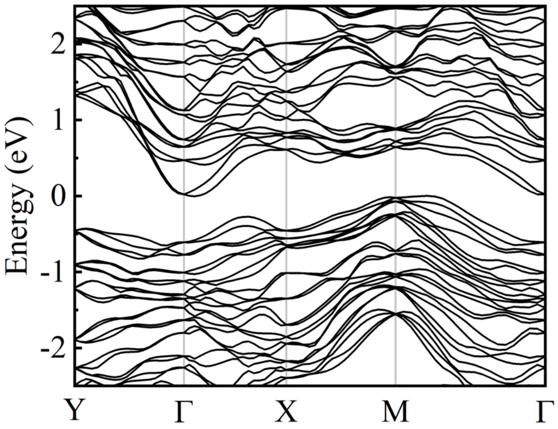

Figure S8. Band structure of FL β-Te under compressive strain of -21% along the NM direction.

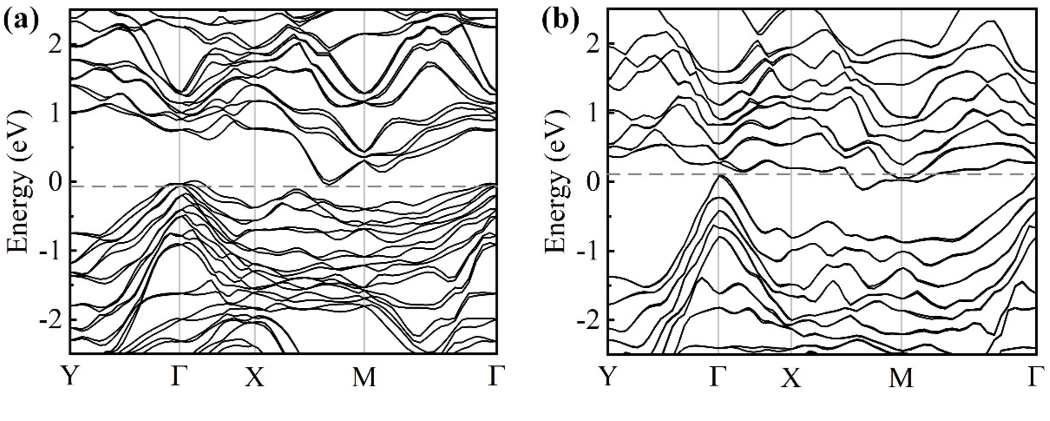

Figure S9. Band structures of metallic TL α-Te induced by uniaxial strains. (a) tensile strains of 11% along AC direction; (b) compressive strains of -8% along ZZ direction.

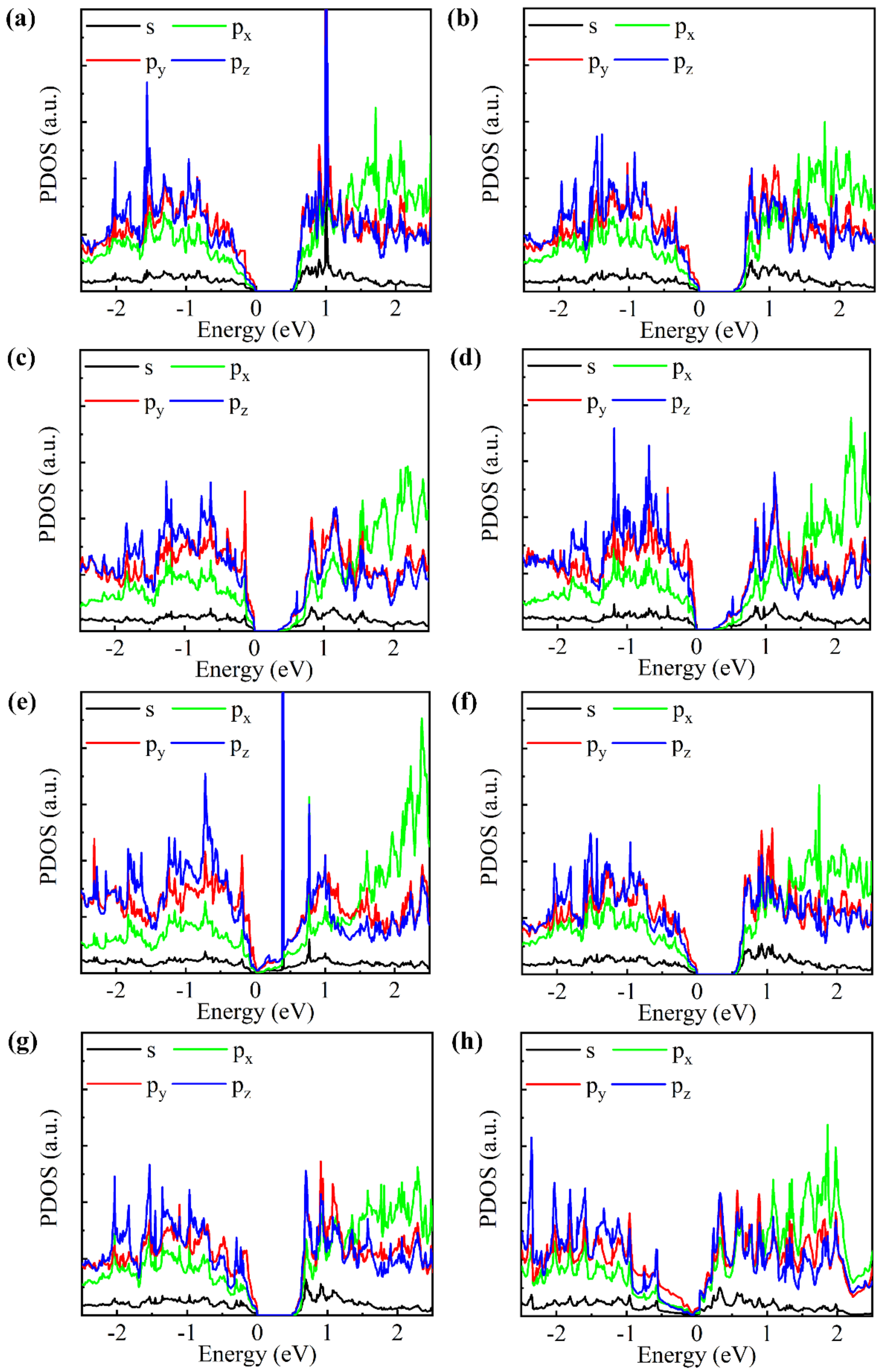

Figure S10. PDOS of TL α-Te under uniaxial strain. (a)Unstrained; The tensile strains of 1%(b), 5%(c), 7% (d) and 11% (e) along the AC direction; The compress strains of -1%(f), -3%(g) and -8%(h) along the ZZ direction.

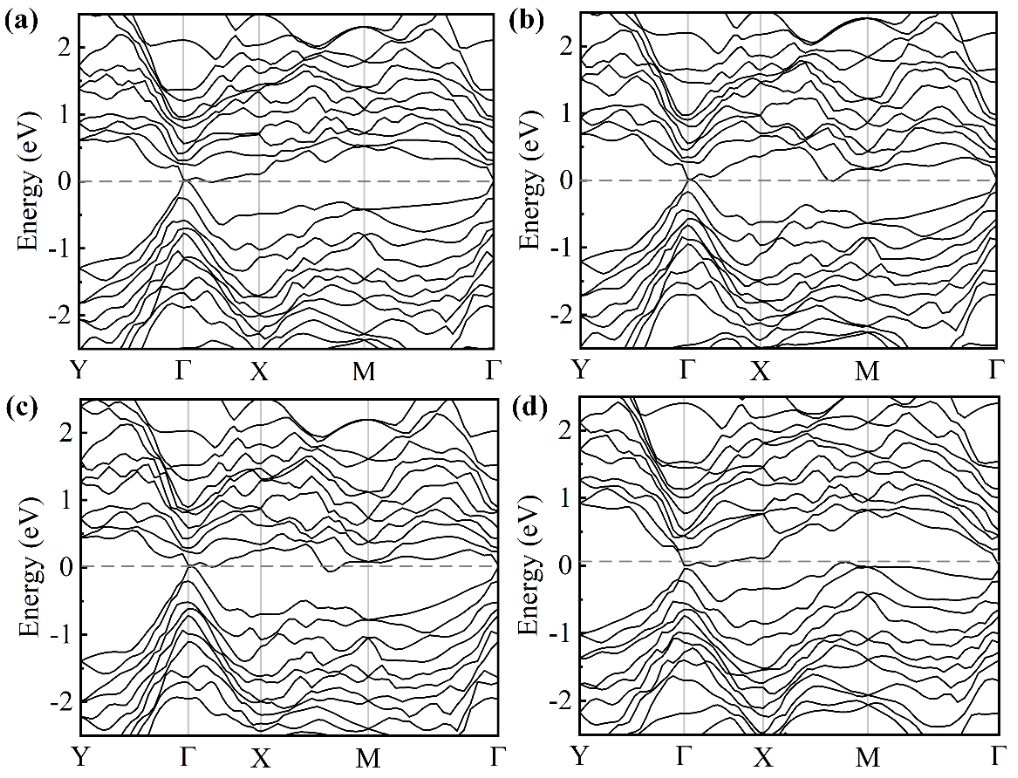

Figure S11. Band structures of metallic FL β-Te induced by uniaxial strain. The strain of -2% (a) and 3% (b) along the AC direction. The strain of -3%(c) and 5% (d) along the ZZ direction.

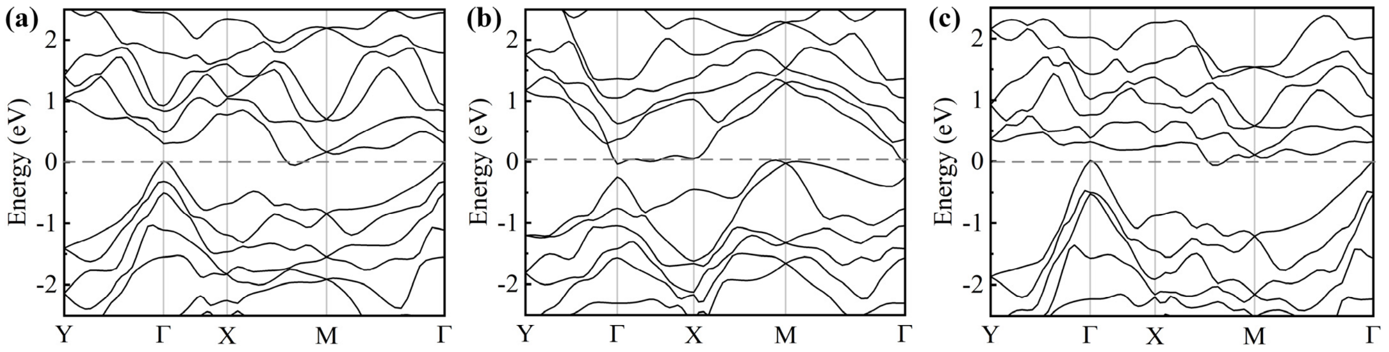

Figure S12. Band structures of metallic BL β-Te induced by uniaxial strain. (a) The tensile strains of 10% along the AC direction; (b) The tensile strains of 10% along the ZZ direction; (c) The compress strains of -7% along the ZZ direction.

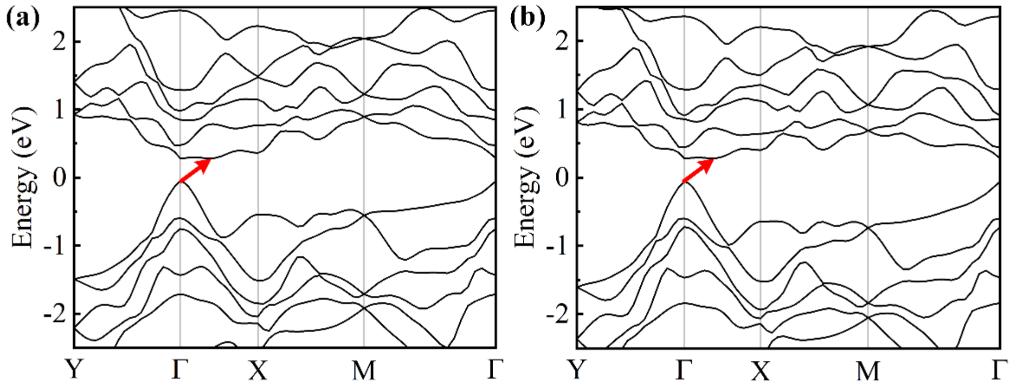

Figure S13. Band structure of BL β-Te under small uniaxial strain along the ZZ direction. (a) 1 %; (b) -1 %.

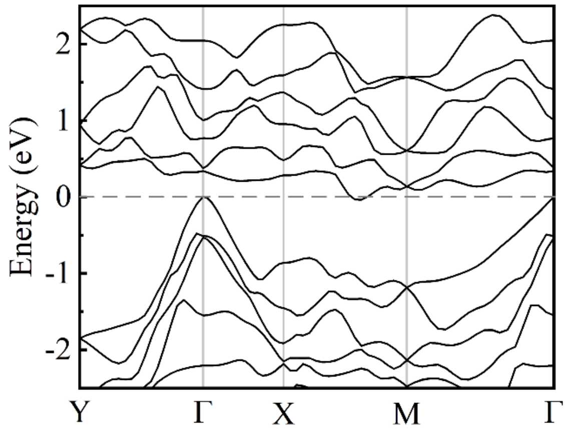

Figure S14. Band structure of BL α-Te under compressive strain of -8% along the ZZ direction.

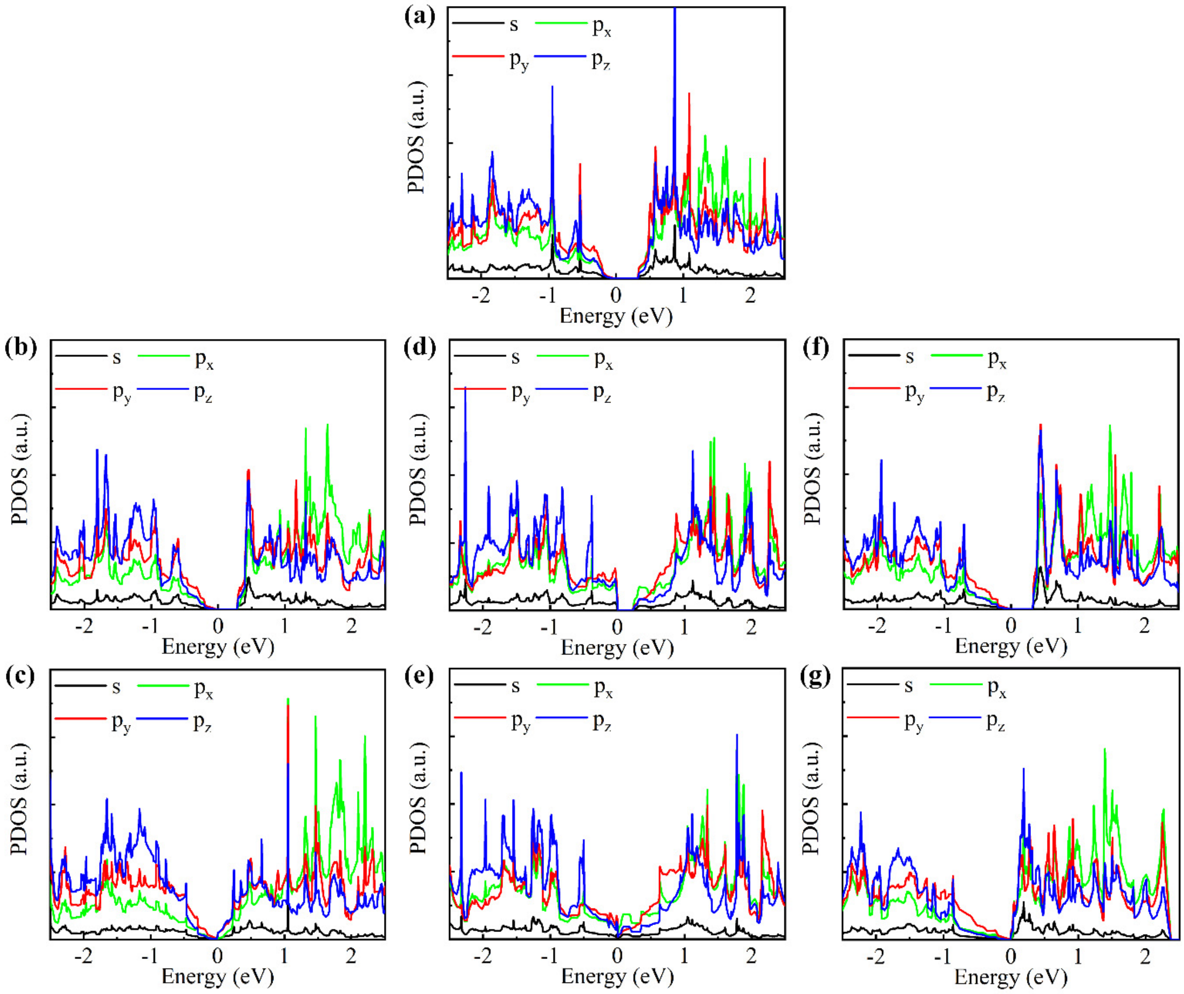

Figure S15. PDOSs of BL of β-Te under uniaxial strain. (a)Unstrained; The tensile strains of 5% (b) and 10% (c) along the AC direction; The tensile strains of 7% (d) and 10% (e) and the compress strains of -3% (f) and -7% (g) along the ZZ direction.

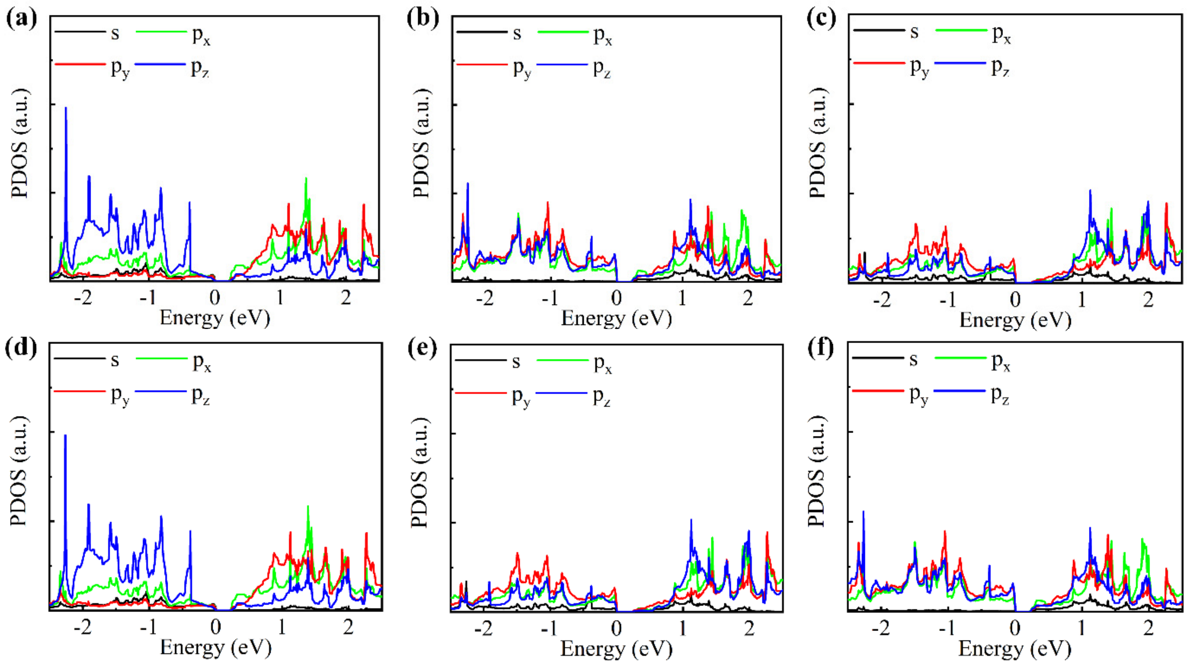

Figure S16. PDOS of Te atoms in BL β-Te under tensile strains of 7% along the ZZ direction. (a) Te_1; (b) Te_2; (c) Te_3; (d) Te_4; (e) Te_5; (f) Te_6.
